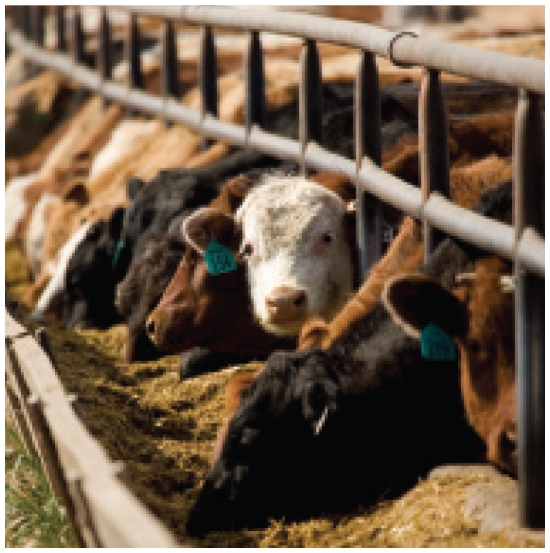# The Beat

**Published:** 2010-05

**Authors:** Erin E. Dooley

## Immunity Insight: Breastmilk and Allergens

A study by Valérie Verhasselt in a supplement to the February 2010 issue of *The Journal of Pediatrics* yields new insights into how breastmilk helps program the immune system of offspring. The milk of lactating mice exposed to the allergen ovalbumin contained ovalbumin and the immune factor TGF-β. Offspring of these mice exposed as adults to ovalbumin were less likely to show symptoms of asthma if their dams had been exposed to the allergen during lactation. The combination of allergen and immune factor in milk appears to be key to producing the protective effect.

## Nzu: From Remedy to Malady?

In December 2009 the FDA issued a national warning advising pregnant and breastfeeding women to avoid consuming *nzu*, a West African traditional remedy for morning sickness sold in pellet or powder form around the world. That warning was based on findings from Texas that samples of *nzu* contained high levels of arsenic and lead. In March 2010 the Guilford County (NC) Department of Public Health also found high levels of lead in samples of *nzu* (60–80 times the FDA limit), prompting a statewide warning. The remedy also may be called calabash chalk, calabar stone, la craie, argile, or mabele.

**Figure:**
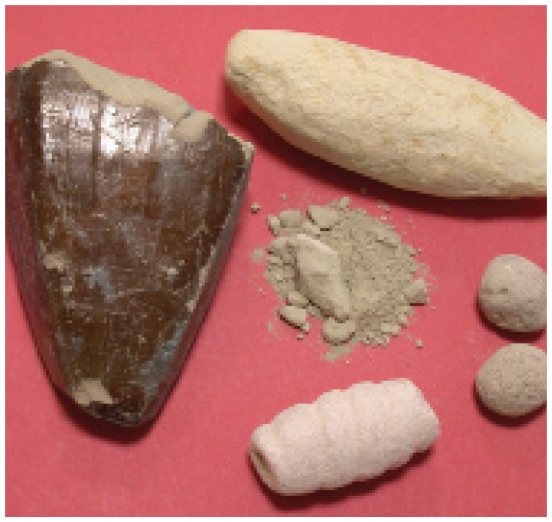
*Nzu* is sold in several different forms

## Improving Predictions of Climate Change Impacts

In March 2010 the National Science Foundation, along with the Departments of Agriculture and Energy, announced a new 5-year interdisciplinary program to develop high-resolution models for predicting climate change and its associated impacts at a local scale. The program, which received about $49 million in funding for its first year, is expected to provide models at significantly improved geographic and temporal resolutions that will be able to help decision makers plan strategies for adapting to the health, ecological, economic, and social changes that could result from a rapidly changing climate.

## High TB Rates among the Inuit

A 10 March 2010 news conference in Ottawa, Canada, highlighted findings that the Inuit population of Canada was infected with tuberculosis at more than 30 times the Canadian national average in 2008. Speakers at the conference, who represented Inuit governing agencies, focused on environmental factors including overcrowded housing and lack of clean drinking water and affordable nutritious food as primary factors in the disparity; many Inuit communities also lack access to quality medical care. They called on the Canadian government to develop a national strategy specific to the Inuit that provides culturally relevant solutions that address living conditions for Canadian Inuit.

**Figure:**
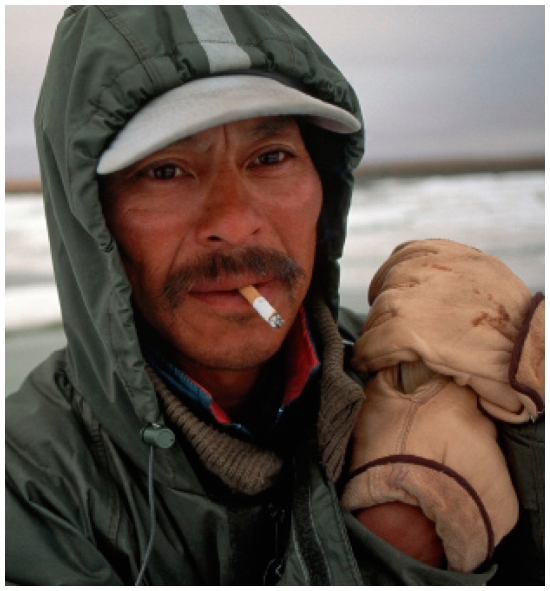


## e-Waste Laws for India

By early May 2010, the Indian Ministry of Environment and Forests expects to approve rules putting responsibility for the disposal of Indian-made electronic products on their manufacturers. The rules were proposed in 2009 by a coalition of environmental advocacy groups and the Indian Manufacturers’ Association for Information Technology. India produces more than 300,000 tons of e-waste annually, a figure that may triple by 2020, according to a recent UNEP report. The new regulations would prohibit the cottage industry of dismantling electronics and recovering the valuable metals they contain, but informal recyclers could still find employment by assisting in the collection of e-waste.

## Animals en Masse

*Livestock in a Changing Landscape*, a two-volume report released in March 2010 by a multi-institution collaborative including the FAO, documents how animal production is causing widespread effects on the environment and human health. Livestock worldwide has tripled over the last 30 years. According to the report, 1.7 billion head of livestock currently occupy more than one-fourth of the land on Earth, and one-third of the Earth’s arable land is devoted to crops used to feed these herds. The report reviews several options for more sustainable animal production. “We want people engaged in the livestock industry to look closely at the report and determine what improvements they can make,” said report co-editor Harold Mooney.

**Figure:**